# Cadherin 23 is a prognostic marker of pancreatic cancer and promotes cell viability in floating culture conditions

**DOI:** 10.7150/ijms.98252

**Published:** 2025-01-01

**Authors:** Chaohui Zhen, Peiwei Sun, Rui Liang, Shuang Fei, Xu Chen, Junlin Qian, Baoshi Xu, QiRui Lin, Guojun Yao, Biao Zheng

**Affiliations:** 1 Department of Surgery, The First Dongguan Affiliated Hospital, Guangdong Medical University. Dongguan, Guangdong 523710, China.; 2 Department of General Surgery, Shenzhen University General Hospital/Shenzhen University Clinical Medical Academy, Shenzhen, Guangdong 518055, China.; 3 Carson International Cancer Research Centre, Shenzhen University School of Medicine, Shenzhen, Guangdong 518055, China.; 4 Department of Surgery and Oncology, Graduate School of Medical Sciences, Kyushu University, Fukuoka, Japan.; 5 Department of Clinical Application, Center for iPS Cell Research and Application (CiRA), Kyoto University, Kyoto, Japan.; 6 Department of Geriatrics, The First Affiliated Hospital of Shenzhen University, Shenzhen Second People's Hospital, Shenzhen, Guangdong 518000, China.

**Keywords:** Pancreatic neoplasms, Cadherin 23, Prognostic marker, Tumor microenvironment

## Abstract

**Purpose**: Pancreatic cancer has the worst prognosis of all common cancers worldwide. Cadherin plays important roles in cancer cell invasion and metastasis. This study investigated the role and mechanism of Cadherin 23 (CDH23) action in the viability of pancreatic cancer cells.

**Methods**: We examined CDH23 expression in 70 surgical pancreatic cancer samples and examined relationships among the level of CDH23 expression, clinicopathological characteristics, and the prognosis of the pancreatic cancer patients. Furthermore, we silenced CDH23 expression in pancreatic cancer cell lines (Panc-1, SUIT-2, MIA PaCa-2, CFPAC-1, and Capan-2) and assessed the viability of these cells. CDH23 expression in pancreatic cancer patients and cell lines was examined using immunohistochemistry and western blotting.

**Results**: High levels of CDH23 in pancreatic cancer patients led to shorter overall survival and correlated with local recurrence and distance metastasis. The viability of pancreatic cancer cells in floating culture conditions decreased sharply when CDH23 was silenced. The viability and migration of pancreatic cancer cells in monolayer culture conditions did not change when CDH23 was silenced. The level of phosphorylated AKT was significantly decreased in the CDH23 knockdown cells in floating culture conditions.

**Conclusion**: High levels of CDH23 expression are correlated with a poor prognosis in pancreatic cancer and may serve as a novel prognostic marker.

## Introduction

Metastasis is a feature of cancer and the major cause of cancer related death 1. Survival of cancer cells once they have detached from the primary tumor is important for the formation of metastases 2; however, the circulation system is a hostile environment and only 0.01% of circulating tumor cells (CTCs) survive to achieve metastasis 3, 4. Cell-cell adhesiveness of metastatic cells allows them to form multicellular clusters within the bloodstream, which increases the survival of such CTCs and contributes to the metastatic spread of cancer 5. Studying the ability of tumor cells to aggregate and maintain their cohesion as they survive in the bloodstream may identify a novel and potentially targetable step in the blood borne dissemination of cancer 5.

Metastatic tumor cells often show changes in multiple cell surface molecules, including cadherins, such as E-cadherin and N-cadherin. These adhesion proteins play important roles in cancer cell invasion and metastasis processes 6-8. Cadherin 23 (CDH23), a member of the cadherin superfamily, is an important constituent of the hair cell tip link. It regulates mechanoelectrical transduction and mutations in *CDH23* cause deafness and age-related hearing loss 9. Mutations in *CDH23* are also detected in pituitary adenoma and play important roles in its pathogenesis 10. CDH23 participates in cell adhesion and its expression is upregulated in breast cancer cells 11, 12, and the strong propensity of CDH23 for aggregation inhibits cell migration in lung adenocarcinoma cells 13. In addition, methylated reduction of CDH23 represents poor outcome of diffuse large B-cell lymphoma 14. Therefore, CDH23 may play a critical function in cancer progression; however, its specific role and mechanism of action are unclear.

In this study, we investigated a potential function for CDH23 in pancreatic tumors by assessing CDH expression in pancreatic ductal adenocarcinoma (PDAC) cell lines and tissue samples. CDH23 upregulated the viability of cancer cells in suspension culture, and this action may involve AKT pathway activation. Clinicopathological analysis indicated CDH23 to also be involved in metastasis and prognosis of PDAC patients.

## Materials and methods

### Cells and culture conditions

The following five pancreatic cancer cell lines were used: CFPAC-1, SUIT-2, MIA PaCa-2 (Japanese Cancer Resource Bank, Tokyo, Japan), Panc-1 and Capan-2 (American Type Culture Collection, Manassas, USA). All cancer cells were maintained in Dulbecco's modified Eagle's medium (DMEM) (Sigma Chemical Co., St. Louis, MO) supplemented with 10% fetal bovine serum (FBS) at 37°C with humidified 90% air and 10% CO_2_. The human immortalized pancreatic ductal epithelial cell line, HPDE6-E6E7 clone 6, was cultured in keratinocyte serum-free media (Gibco) supplemented with epidermal growth factor (5 ng/ml), bovine pituitary extract (50 µg/ml), and penicillin-streptomycin (Gibco). Floating culture was performed using Ultra-Low attachment surface plates (Corning, USA).

### Pancreatic tissues

We analyzed CDH23 expression in 70 tissue samples obtained from patients who underwent pancreatic resection for pancreatic cancer at our institution (Department of Surgery and Oncology, Graduate School of Medical Sciences, Kyushu University) between January 2005 and August 2012. We also obtained normal pancreatic tissue samples from intact pancreas resected during bile duct cancer surgery as control tissues. Survival was measured from the time of pancreatic resection, with death as the end point. Overall survival and disease-free survival analyses were performed in February 2016. The median observation time for overall survival and disease-free survival was 19 months (range 2-137 months) and 11 months (range 1-137 months), respectively. Forty-nine patients died during the follow-up. All surviving patients were followed up. Histological diagnosis of specimens was in accordance with the criteria of the updated World Health Organization classification 15. Tumor stage was assessed according to The Union for International Cancer Controls (UICC) classification, 7th edition 16. The study was approved by the Ethics Committee of the First Dongguan Affiliated Hospital, Guangdong Medical University and conducted according to the Ethical Guidelines for Human Genome/Gene Research enacted by the Japanese Government and the Helsinki Declaration.

### Immunohistochemical procedures and evaluation

Immunohistochemistry was performed for CDH23 using a Histofine SAB-PO kit (Nichirei, Tokyo, Japan) as described previously 17. Endogenous peroxidase activity was blocked with methanol containing 0.3% hydrogen peroxidase. Antigen retrieval was performed by boiling in a microwave oven (in citrate buffer, pH 6.0). Sections were incubated with an anti-CDH23 antibody (H00064072-A01, Abnova, Taipei, Taiwan) overnight at 4°C. Carcinoma cells were identified according to morphology and counted in at least 20 fields per section at 200 × magnification. The distribution of CDH23 staining was evaluated as the percentage of stained cells, and was scored as follows: 0, no staining or less than 10%; 1, 11%-25%; 2, 26%-50%; 3, 51%-75%; and 4, 76%-100%. Cells were also scored for staining intensity, which was scored as 0, no staining; 1, weak; 2, moderate; or 3, strong. The multiplication product from these two scores (from 0-12) was used to assign patients into one of two groups according to CDH23 expression; a score of 0-4 represented low expression and a score of 6-12 represented high expression.

### Silencing CDH23 using small interfering RNAs (siRNAs)

Gene silencing was performed using siRNA (Qiagen, MA, USA) directed against human *CDH23*. Target sequences were: siRNA-1 (5′-CCCAAATGTGTGCCCAGCTTA-3′); siRNA-2 (5′-CAGCGGAGTGCTGACCTTGAA-3′). Qiagen all-star siRNA was used as a negative control. Transfections were performed as described previously 18. All cells were used in subsequent experiments 48 h after transfection.

### Western blotting analysis

Western blotting was performed as described previously 17. Cells were lysed in PRO-PREP (iNtRON Biotechnology, Seongnam, Korea) and proteins were separated on 4%-15% Mini-PROTEAN TGX Precast Gels (Bio-Rad Laboratories) and transferred to Trans-Blot Turbo Mini PVDF Transfer Packs (Bio-Rad Laboratories) using a Trans-Blot Turbo Transfer Starter System (Bio-Rad Laboratories). Antibodies used in this study were: anti-CDH23 (H00064072-A01, Abnova, Taipei, Taiwan), anti-E-cadherin (# 3195), anti-AKT (# 4691), anti-Phospho-AKT (# 4060), (Cell Signaling Technology, Danvers, MA, USA) and anti-GAPDH (ab8245; Abcam, Cambridge, MA, USA). Immunoblot signals were detected by enhanced chemiluminescence with ChemiDoc XRS (Bio-Rad Laboratories).

### Migration assays

Migration of cultured cancer cells was assessed by counting the number of cells migrating through Transwell chambers (BD Biosciences, Franklin Lakes, NJ) as described previously 18. Cells were maintained in 10% FBS/DMEM during these assays. Cells were transfected with siRNAs 48 h prior to the assay. Migration was determined after a 24 h period.

### Floating culture system

Cells were seeded in a 96-well plate with an ultra-low attachment surface and round bottom (Product Number 7007; Corning Inc., Corning, NY, USA,). Photomicrographs of cells were taken using a BIOREVO BZ9000 microscope (Keyence, Osaka, Japan) and areas of spheroids were measured using a BZ Analyzer (Keyence).

### Cell Viability Assay

Pancreatic cells (1.5 × 10^3^ cells/well) were seeded in 96-well plates (Greiner Bio-One, Frickenhausen, Germany) or Ultra-Low attachment surface 96-well plates (#7007, Corning, USA) 24 h after transfection with siRNA. Cell viability was examined using the CellTiter-Glo Luminescent Cell Viability Assay Kit (G7570, Promega) following the manufacturer's instructions. Background was subtracted using values from wells containing only culture medium.

### Caspase-3 activity assay

SUIT-2 cells (2.0 × 10^4^ cells/well) were seeded in 6-well culture plate or Ultra-Low attachment surface 6-well plates (#3471, Corning, USA) 24 h after transfection with siRNA. Protein extracts were prepared following manufacturer^'^s instructions by using Bradford Protein Assay kit (Beyotime Institute of Biotechnology, Nantong, Jiangsu, China). Caspase-3 activity was measured using Caspase-3 Activity Assay kit (Beyotime Institute of Biotechnology, Nantong, Jiangsu, China) in which cell extracts were mixed with Ac-DEVD-pNA substrate for 2 h at 37°C in 96-well plates prior to colorimetric measurement of p-nitroanilide product at 405 nm 19.

### Statistical analysis

A χ2-test was used to analyze the correlation between CDH23 expression and clinicopathological characteristics. Survival analysis was performed using Kaplan-Meier analysis and curves were compared using the log-rank test. For *in vitro* experiments, values are expressed as the mean ± standard deviation. Comparison between two groups was performed using Student's t-test. Statistical significance was defined as *P* < 0.05. All statistical analyses were performed using JMP 13 software (SAS Institute, NC, USA).

## Results

### High CDH23 expression is significantly associated with poor prognosis

We analyzed general data and prognostic factors of 70 patients undergoing pancreatic cancer surgery. Their clinicopathological characteristics are listed in **Table 1**. The results of univariate analysis showed that better 5-year overall survival of patients was correlated with low CDH23 expression, early tumor stage, no residual tumor, early histological grade and negative vascular invasion (*P* < 0.05) **(Table 2)**. The results of multivariate analysis showed that CDH23 expression and residual tumor were adverse factors for overall survival **(Table 3)**. These results indicated that high CDH23 expression was associated with poor prognosis of pancreatic cancer.

### CDH23 expression promotes pancreatic cancer metastasis and recurrence

**Figure 1:**
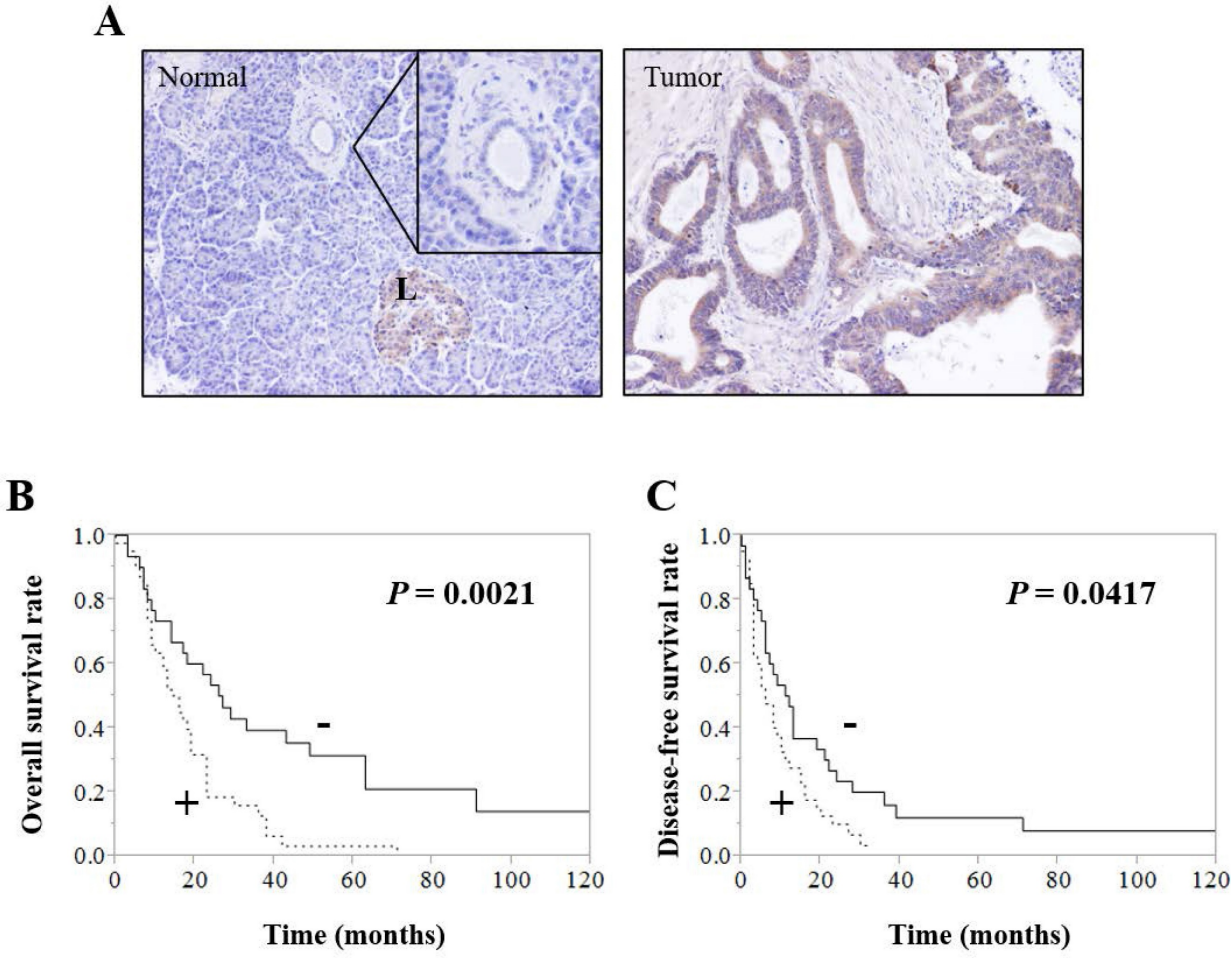
** CDH23 expression correlates with poor outcome in pancreatic ductal adenocarcinoma. (A)** Immunohistochemical analysis of CDH23 in human pancreatic ductal adenocarcinoma (PDAC) specimens. A normal pancreatic specimen showing weak-to-no staining in pancreatic ductal epithelial cells. **(B)** And **(C)** Kaplan-Meier survival analysis of CDH23 expression in PDAC cancer cells. High CDH23 expression was associated with shorter overall survival (B) and disease-free survival (C).

Immunohistochemistry showed that CDH23 was expressed in normal pancreatic tissues of control patients and tumor tissues of pancreatic cancer patients; however, the level of CDH23 expression in pancreatic cancer patient tumor tissues was significantly higher compared with normal pancreatic tissues of control patients** (Fig. 1A)**. CDH23 expression was strongly associated with local recurrence and distant metastasis** (Table 4)**. Moreover, Kaplan-Meier analysis indicated that pancreatic cancer patients with high levels of CDH23 expression suffered from poor survival** (Fig. 1B/C)**.

### Inhibition of CDH23 expression does not affect migration or viability of pancreatic cancer cells in monolayer culture conditions

**Figure 2:**
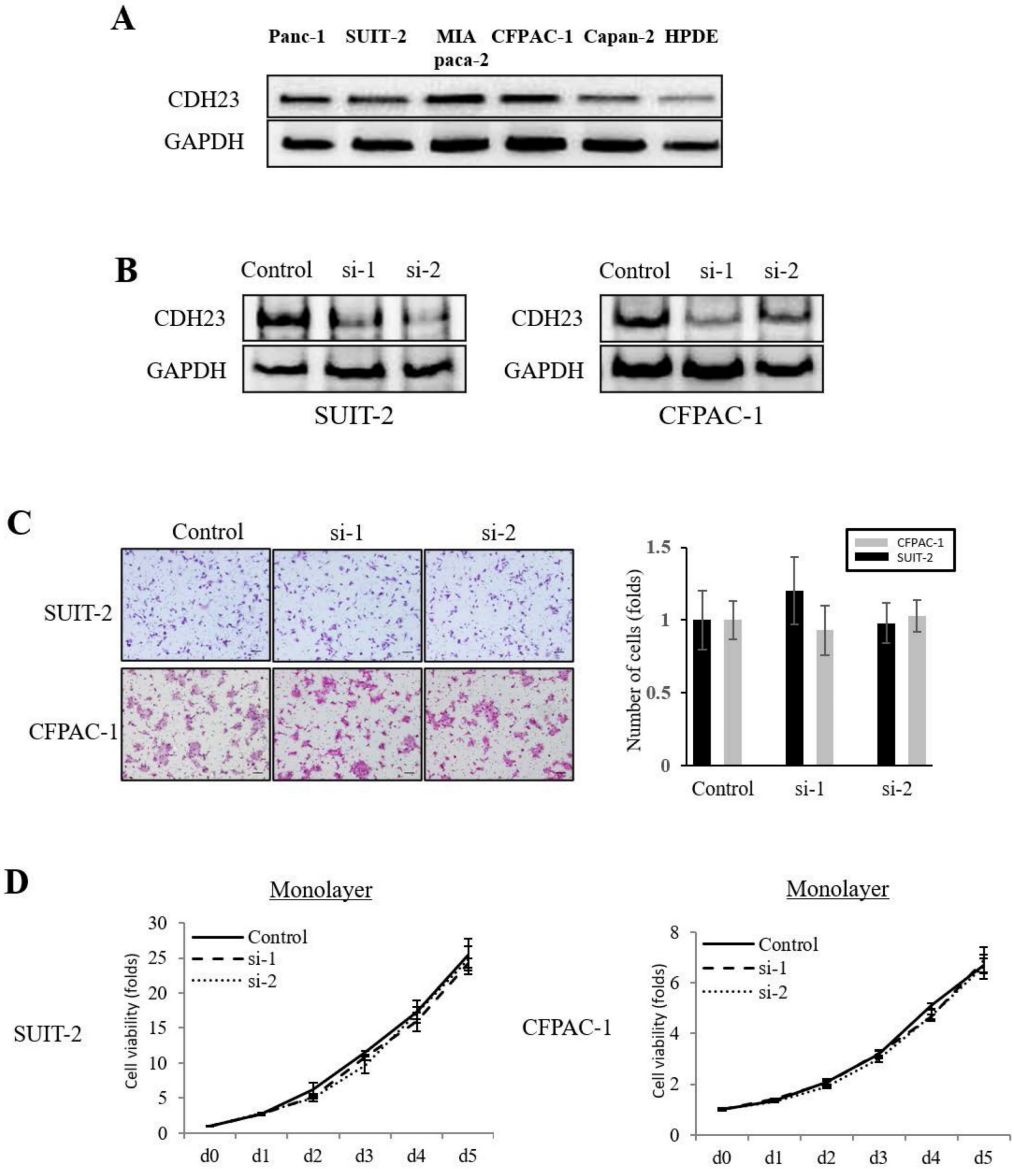
** Knockdown of CDH23 does not affect migration or viability of pancreatic cancer cells in monolayer culture conditions. (A)** Representative western blot analysis of CDH23 in distinct pancreatic cancer cells. GAPDH abundance is used as an internal control. Cell types, protein masses, and siRNA treatment are indicated. **(B)** A stable cell line with CDH23 knocked-down was generated using siRNA. Down-regulation of CHD23 was confirmed by western blotting. GAPDH was used as a control. **(C)** Evaluation of SUIT-2 and CFPAC-1 cell migration following transfection with control or CDH23-specific siRNAs. Representative photomicrographs are shown in the panels on the left-hand side. Bar charts summarize the migration of cells in each siRNA treatment group. **(D)** Evaluation of SUIT-2 and CFPAC-1 cell viability following transfection with control or CDH23-specific siRNAs in monolayer culture conditions. Line charts show the viability of cells in each siRNA treatment group in monolayer culture conditions.

The expression of CDH23 in pancreatic cancer cell lines (Panc-1, SUIT-2, MIA PaCa-2, CFPAC-1, Capan-2) was higher than that in normal pancreatic epithelial cells (HPDE cells)** (Fig. 2A)**. To investigate the effect of CDH23 expression in pancreatic cancer cells, we stably downregulated CDH23 expression by siRNA in SUIT-2 and CFPAC-1 cells and confirmed downregulation at both mRNA and protein levels **(Fig. 2B)**. Furthermore, Transwell chamber assays showed that CDH23 silencing did not inhibit migration or cell viability of pancreatic cancer cells in monolayer culture conditions **(Fig. 2C/D)**.

### CDH23 promotes viability of pancreatic cancer cells in floating culture conditions

**Figure 3:**
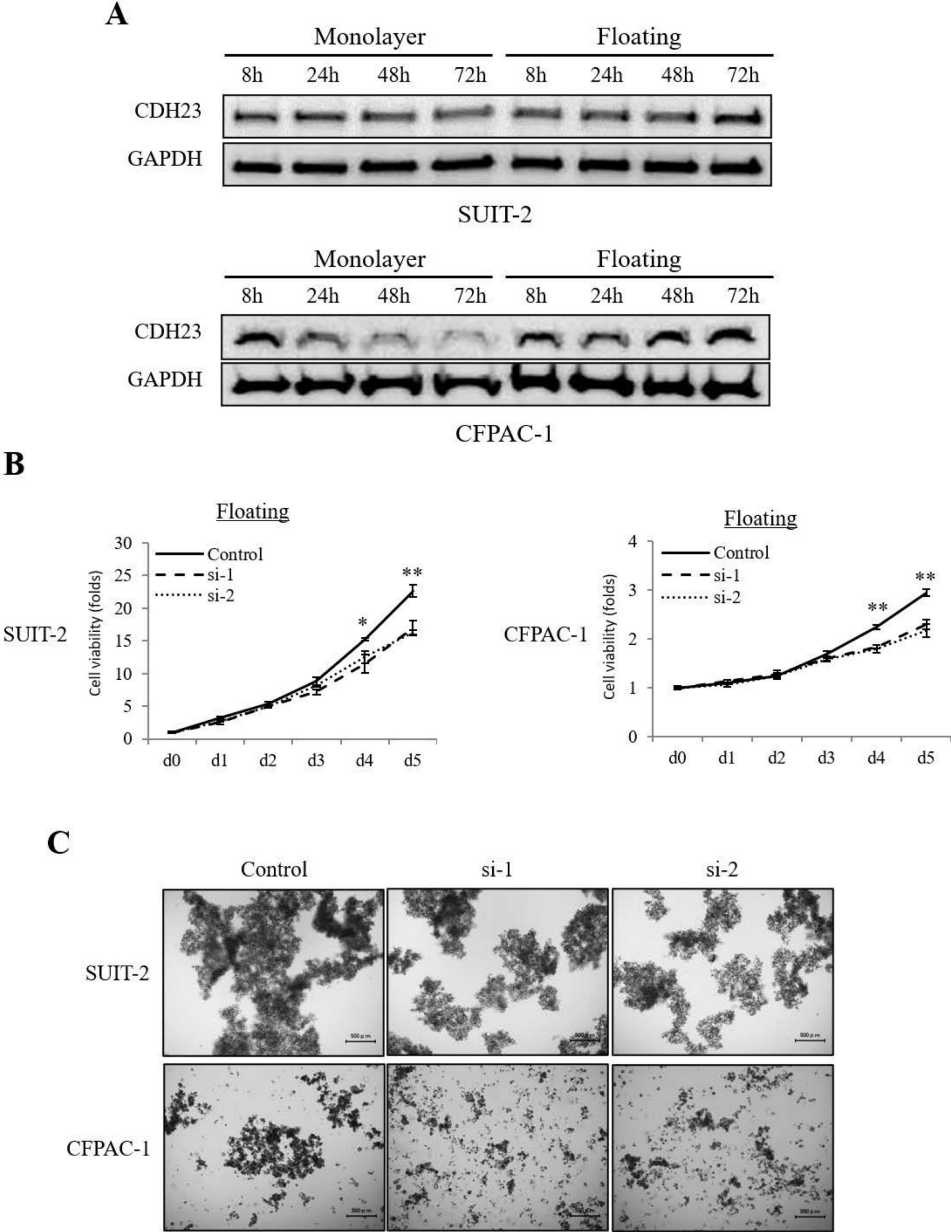
** CDH23 expression promotes viability of pancreatic cancer cells in floating culture conditions. (A)** Western blotting showing CDH23 protein levels in SUIT-2 and CFPAC-1 pancreatic cancer cells at 8, 24, 48 and 72 h in monolayer and floating culture conditions. **(B)** Evaluation of the viability of SUIT-2 and CFPAC-1 cells following transfection with control or CDH23-specific siRNAs in floating culture conditions. Line charts show the viability of cells in each siRNA treatment group in floating culture conditions. (* *P* < 0.05; ** *P* < 0.01)** (C)** Representative images of SUIT-2 and CFPAC-1 cell cluster following transfection with control or CDH23-specific siRNAs in floating culture conditions.

Interestingly, the expression of CDH23 was higher in floating culture conditions than in monolayer culture conditions **(Fig. 3A)**. When CDH23 expression was downregulated in pancreatic cancer cells by siRNA, their viability in floating culture conditions decreased sharply **(Fig. 3B)**, and high CDH23 expression seems more likely to form cell clusters **(Fig. 3C)**.

### CDH23 promotes AKT phosphorylation in pancreatic cancer cells in floating culture conditions

**Figure 4:**
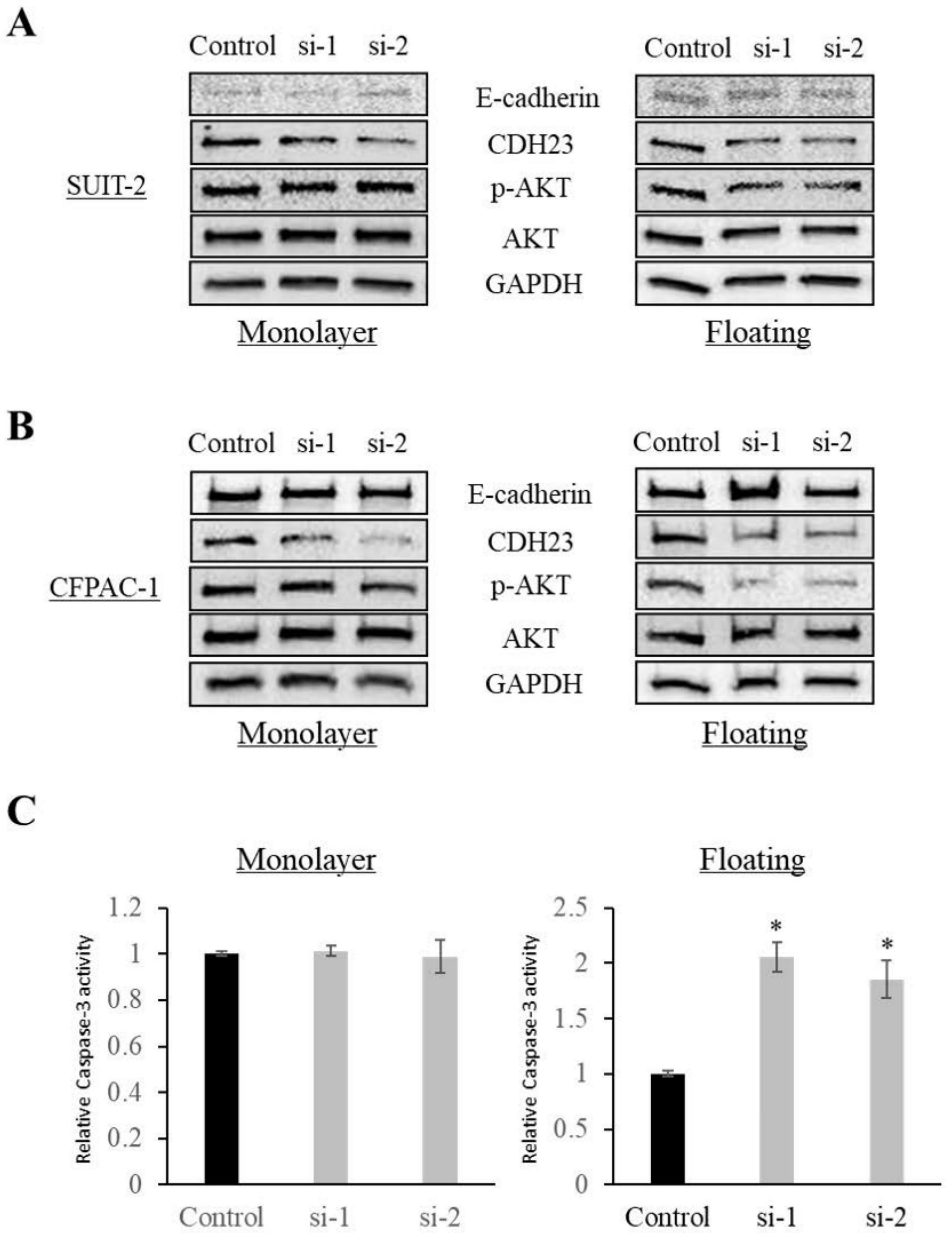
** CDH23 expression promotes AKT phosphorylation in floating culture conditions. (A)** Phosphorylation of AKT was reduced after knockdown of CDH23 in SUIT-2 cells in floating culture conditions. No remarkable change was observed in monolayer culture conditions. **(B)** Phosphorylation of AKT was reduced after knockdown of CDH23 in CFPAC-1 cells in floating culture conditions. No remarkable change was observed in monolayer culture conditions. **(C)** Caspase-3 activities was increased after knockdown of CDH23 in floating culture conditions. No remarkable change was observed in monolayer culture conditions. (* *P* < 0.05)

Western blotting showed that CDH23 knockdown increased the expression of E-cadherin and decreased the phosphorylation of AKT in cells in floating culture conditions (*P* < 0.05), while there was no significant difference in monolayer culture conditions (**Fig. 4A, B**). In addition, knockdown of CDH23 increased Caspase-3 activity under floating culture conditions, but CDH23 knockdown does not affect the pro-apoptotic protein Caspases-3 under monolayer culture conditions (**Fig. 4C**).

## Discussion

Pancreatic cancer is a malignant digestive system tumor with insidious onset, invasion and early-stage metastasis. Although the existing treatment methods continue to advance, the overall prognosis of patients is still very poor. In recent years, the incidence of pancreatic cancer has increased worldwide. High rates of invasion and metastasis are independent risk factors affecting the prognosis of patients with pancreatic cancer. Therefore, it is important to further clarify the molecular mechanisms associated with invasion and metastasis of pancreatic cancer and to find marker genes that can guide early diagnosis and treatment of patients.

CDH23 is a cadherin that is widely distributed on the cell membrane surface of normal cells. It is also present in many tumors, including breast, colorectal, and renal cancer tissues 9, 20, 21, and upregulation of CDH23 expression corresponds to a decrease of overall survival in patients with acute myeloid leukemia 22. Our clinical data showed a poorer prognosis for pancreatic cancer patients with high levels of CDH23 expression, and CDH23 expression was associated with local recurrence and distant metastasis. These data suggest that high CHD23 expression in pancreatic cancer tissues is related to progression of the disease and may be a marker for the prognosis of pancreatic cancer.

The metastatic ability of tumor cells is an important feature of malignant tumors that affects the recurrence and metastasis of tumors. Moreover, circulating tumor cell (CTC) clusters contribute to metastatic propensity, especially when they affect the ability of epithelial tumor cells to survive the loss of cell adherence and shear forces in the blood stream 23. In this context, either mesenchymal transformation, stromal-derived factors, or persistent interepithelial cell junctions may provide survival signals that attenuate this apoptotic outcome 23. CTC clusters are more likely to resist initial cell death after lodging in distant organs, thereby promoting metastasis 24. In this study, we found that CHD23 promotes pancreatic cancer cells viability under the floating culture conditions but not under the monolayer culture conditions, and high CDH23 expression is more likely to promote the formation of cell clusters. CDH23 is also upregulated in breast cancer cells, and it participates in cell adhesion and play a role in the early stages of metastasis 11. Therefore, CDH23 may affect the viability of floating cells by regulating cell adhesion, thus promoting the metastasis and progression of pancreatic cancer.

This study also analyzed the molecular mechanism of CDH23 action on pancreatic cancer cell lines. Knockdown of CDH23 significantly decreased the level of Akt activation, while it increased the activity of pro-apoptotic factor Caspase-3, both are in the floating culture conditions. The Akt signaling regulates cell proliferation and survival, however, Akt regulation of cell survival involves direct inhibition of pro-apoptotic signals 25. Therefore, CDH23 may promote the viability of pancreatic cancer cells through Akt signal. Pancreatic cancer cells survive after leaving the extracellular matrix under floating culture conditions, partly by inhibiting cell apoptosis. The specific molecular mechanism needs to be further clarified.

## Conclusion

In this study, we detected the expression of CDH23 in pancreatic cancer and explored the impact of CDH23 on tumor development. We found that the expression of CDH23 affects the progression and prognosis of pancreatic cancer. In addition, the CDH23 signaling pathway regulates the phosphorylation of Akt and affects cell viability under floating culture conditions. Therefore, these data provide new insights into the pathogenesis of pancreatic cancer, and CDH23-mediated signal transduction may be a new therapeutic target.

## Figures and Tables

**Table 1:** Clinicopathological characteristics of patients (n=70)

Median age	67 years (Range 36-85 years)
Sex (Male/Female)	46(65.7%) / 24 (34.3 %)
pT category	
T1	3(4.3%)
T2	1(1.4%)
T3	64(91.4%)
T4	2(2.9%)
pN category	
pN0	17 (24.3%)
pN1	53(75.7%)
UICC stage	
I	4(5.7%)
II	62(88.6%)
III	1(1.4%)
IV	3(4.3%)
Residual tumor category	
R0	52(74.3%)
R1	18(25.7%)
Histologic grade	
Grade 1	15(21.4%)
Grade 2	31(44.3%)
Grade 3	24(34.3%)
Vascular invasion	
Negative	26(37.1 %)
Positive	44(62.9 %)
Perineural invasion	
Negative	11(15.7%)
Positive	59(84.3%)
Lymphatic invasion	
Negative	14(20.0%)
Positive	56(80.0%)

**Table 2:** Univariate survival analysis of conventional prognostic factors and CDH23 expression in pancreatic cancer patients resection (n = 70)

Characteristics	No. of cases	Median Survival time (Mo)	5 -Year Survival Rate (%)	*P* value
CDH23 expression				0.0021
Low	30	26.5	28.4	
High	40	16	4.4	
Age				0.6863
< 65	28	19	12.1	
≥ 65	42	17	15.8	
Sex				
Male	46	23	12.5	0.3658
Female	24	22	17.3	
pT category				0.3309
pT1/ pT2	4	35.5	37.1	
pT3/ pT4	66	18	12.1	
pN category				0.0762
pN0	17	24	31.5	
pN1	53	16	8.8	
UICC stage				0.0091
I / II	66	19	15.4	
III / IV	4	8.5	0.18	
Residual tumor				0.0002
R0	52	23	18.9	
R1	18	9	1.6	
Histologic Grade				0.0406
Grade 1	15	31	33.1	
Grade 2	31	19	12.0	
Grade 3	24	9.5	4.9	
Lymphatic invasion(ly)				0.5055
Negative	14	20	19.4	
Positive	56	18	12.6	
Vascular invasion (v)				0.0122
Negative	26	27	26.9	
Positive	44	13	6.9	
Perineural invasion (ne)				0.1621
Negative	11	30	28.6	
Positive	51	17	11.0	

**Table 3:** Multivariate analysis of conventional prognostic factors and CDH23 expression in pancreatic cancer patients

Characteristics	Relative Risk		95%Confidence Interval	*P* value
CDH23 expression	2.321	1.307-4.228		0.0038
Residual tumor(R1)	2.568	1.240-5.192		0.0119
UICC stage	1.412	0.386-4.073		0.5695
Histologic Grade	-	-		0.6193
Vascular invasion	1.237	0.652-2.379		0.5165
				

The relative risks of UICC stage and Histological grade are not shown because of the two parameters involved

**Table 4:** Relationship between CDH23 expression and various clinicopathological factors in patients with pancreatic ductal adenocarcinoma (n = 70)

Characteristics	Low expression group N = 30(42.9%)	High expression group N = 40(57.1%)	*P* value
Age			0.0545
< 65	8(26.67)	20(50.00)	
≥ 65	22(73.33)	20(50.00)	
Sex			0.7166
Female	11(36.67)	13(32.50)	
Male	19(63.33)	27(67.50)	
pT category			0.7673
pT1 / pT2	2(6.67)	2(5.00)	
pT3 / pT4	28(93.33)	38(95.00)	
pN category			0.6881
pNo	8(26.67)	9(22.50)	
pN1	22(73.33)	31(73.50)	
UICC staging			0.4443
I / II	29(96.67)	37(92.50)	
III / IV	1(3.33)	3(7.50)	
Residual tumor			0.6922
R0	23(76.67)	29(72.50)	
R1	7(23.33)	11(27.50)	
Histologic grade			0.6190
Grade 1	8(26.67)	7(17.50)	
Grade 2	13(43.33)	18(45.00)	
Grade 3	9(30.00)	15(37.50)	
Lymphatic invasion(ly)			0.5475
Negative	7(23.33)	7(17.50)	
Positive	23(76.67)	33(82.50)	
Vascular invasion(v)			0.3540
Negative	13(43.33)	13(32.50)	
Positive	17(56.67)	27(67.50)	
Perineural invasion(ne)			0.8499
Negative	5(16.67)	6(15.00)	
Positive	25(83.33)	34(85.00)	
Local recurrence			0.0082
Negative	17(56.67)	34(85.00)	
Positive	13(43.33)	6(15.00)	
Distance metastasis			0.0307
Negative	15(50.00)	10(25.00)	
Positive	15(50.00)	30(75.00)	
